# An Age-Old Problem

**DOI:** 10.1371/journal.pgen.0030037

**Published:** 2007-02-23

**Authors:** Nicholas Katsanis, Susan M Rosenberg

*Contra vim mortis non crescit herba in hortis.*

*“There is no herb in the gardens against the power of death.”* —

King Sigismund III (1566–1632) on his deathbed

Rapid advances in the basic and medical sciences, while rapidly expanding our knowledge base on a host of physiological processes (and their cellular and organismal consequences when disrupted), are also posing a significant burden. The sheer volume of information amassed from a host of experimental systems and model organisms can represent a daunting assimilation task to both the neophyte and the experienced investigator, a problem often compounded by contradictory and/or conflicting data that are inherent to the constant flux of scientific discovery.

With these challenges in mind, we are pleased to introduce a new series initiative by *PLoS Genetics*. In keeping with the mission statement of our journal to present interdisciplinary research in the broadest possible context, we have commissioned a series of Review and Opinion articles bound thematically to a discrete set of topics of inherent complexity, as well as broad interest. Each component of the series will examine a facet of the chosen problem and we hope that the amalgam of each series, which will be available electronically as a unified entity, will both educate the non-specialist as well as provide a balanced view that will transmit to our readership an appreciation of the progress made and the future trends in each field.

Our inaugural series focuses on aging, a field under both rapid evolution and substantial controversy. Humans are arguably unique among sentient species in that we are cognizant of our own mortality even at moments when it is not imminent—a fact that has constituted a major force in shaping our civilization (in all its iterations). The advent of molecular biology and genetics has offered a unique opportunity to help us understand why organisms age, which in turn might offer clues as to how one might decelerate, stop, or even reverse this process. When examining the field of aging research, we can identify three major spheres: cellular aging (senescence/quiescence), organismal aging, and age-related disorders. In this series, we will focus primarily of the first two areas, because the study of age-related disorders does not necessarily inform the basic questions, namely: (a) what are the basic determinants of lifespan, and (b) what are the fundamental cellular and molecular mechanisms that underscore aging processes?

**A Look at the Science behind the Genetics of Aging:**
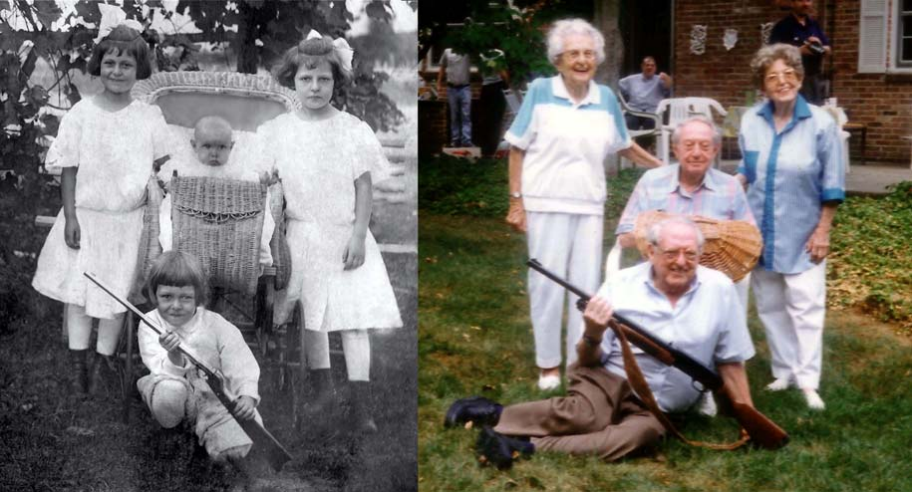
(Image: photograph kindly provided by the Keane family, PLoS Biology 4[4]: e119)

Like most disciplines, aging research has benefited from the availability of a host of prokaryotic and eukaryotic model organisms. However, although some aging mechanisms seem to hold true in diverse species, such as the benefits of caloric restriction in prolonging lifespan, there are also multiple aging mechanisms, and their possible clade specificity has generated potential for controversy. This highlights an important feature of the aging process; aging occurs in many species in nature, and is particularly obvious in captivity where natural hazard is removed. The aging process is in itself, however, nonadaptive and it evolves because natural selection is relatively powerless to act on the traits of older individuals. Nonetheless, the study of both species-specific and ubiquitous aging mechanisms will inform the biological process of aging and will ultimately be better reflected in the management of aging in humans.

We are pleased to introduce the first element of our series in this month's issue, in which Tom Prolla and colleagues will discuss the effect of mitochondrial mutations on the aging process in the mouse [1]. As our discussion progresses, we will bring perspectives that encapsulate work in other species and cross-reference those electronically, accompanied by selected original research papers from the field. Linda Partridge will summarize recent progress in understanding the aging process in *Drosophila,* and a separate review will address the current state of affairs in our understanding of the genetic and epigenetic modulation of healthspan and lifespan in humans (including allied premature aging disorders). In parallel, papers will focus on the aging process in yeast, recent work in the field of C. elegans aging, and recent exciting work in bacterial aging.

We very much hope that our readership will enjoy these articles, appreciate both the depth and breadth of the topic, and find this electronically bound series a useful intellectual and educational tool. 
